# Predicting the retinal content in omega‐3 fatty acids for age‐related macular‐degeneration

**DOI:** 10.1002/ctm2.404

**Published:** 2021-06-30

**Authors:** Niyazi Acar, Bénédicte M. J. Merle, Soufiane Ajana, Zhiguo He, Stéphane Grégoire, Boris P. Hejblum, Lucy Martine, Benjamin Buaud, Alain M. Bron, Catherine P. Creuzot‐Garcher, Jean‐François Korobelnik, Olivier Berdeaux, Hélène Jacqmin‐Gadda, Lionel Bretillon, Cécile Delcourt, for the Biomarkers of Lipid Status And metabolism in Retinal ageing (BLISAR) Study Group, Niyazi Acar, Soufiane Ajana, Olivier Berdeaux, Lionel Bretillon, Alain Bron, Benjamin Buaud, Stéphanie Cabaret, Philippe Gain, Audrey Cougnard‐Grégoire, Catherine Creuzot‐Garcher, Cécile Delcourt, Marie‐Noelle Delyfer, Catherine Féart, Valérie Febvret, Stéphane Grégoire, Zhiguo He, Jean‐François Korobelnik, Lucy Martine, Bénédicte MJ Merle, Gilles Thuret, Carole Vaysse

**Affiliations:** ^1^ Eye and Nutrition Research Group AgroSup Dijon CNRS INRAE Centre des Sciences du Goût et de l'Alimentation Université Bourgogne Franche‐Comté Dijon France; ^2^ ChemoSens Platform Centre des Sciences du Goût et de l'Alimentation AgroSup Dijon, CNRS, INRAE Université Bourgogne Franche‐Comté Dijon France; ^3^ Team LEHA, UMR 1219 Bordeaux Population Health Research Center Univ. Bordeaux, INSERM F‐33000 Bordeaux France; ^4^ Biology, Imaging, and Engineering of Corneal Graft Faculty of Medicine Jean Monnet University, Saint Etienne France; ^5^ Team SISTM, UMR 1219 Bordeaux Population Health Research Center Univ. Bordeaux, INSERM, INRIA BSO F‐33000 Bordeaux France; ^6^ ITERG ‐ Equipe Nutrition – Santé and Biochimie des Lipides Canéjan France; ^7^ Department of Ophthalmology University Hospital Dijon France; ^8^ CHU Bordeaux Service d'ophtalmologie Bordeaux France; ^9^ Centre des Sciences du Goût et de l'Alimentation, AgroSup Dijon, CNRS, INRAE Université Bourgogne Franche‐Comté Dijon France; ^10^ Univ. Bordeaux, INSERM, Bordeaux Population Health Research Center, team LEHA, UMR 1219 F‐33000 Bordeaux France; ^11^ Department of Ophthalmology University Hospital Dijon France; ^12^ ITERG ‐ Equipe Nutrition Santé & Biochimie des Lipides Canéjan France; ^13^ Laboratory for Biology, Imaging, and Engineering of Corneal Grafts, EA2521, Faculty of Medicine University Jean Monnet Saint‐Etienne France; ^14^ CHU de Bordeaux, Service d'Ophtalmologie Bordeaux F‐33000 France

Dear Editor,

Current treatments for age‐related macular degeneration (AMD)―the leading cause of blindness in industrialized countries―are restricted to a single form of the disease and cannot always avoid severe visual loss.^1^ Among emerging preventive strategies, ω‐3 polyunsaturated fatty acids (PUFAs) are enjoying interest as they promote normal retinal structure and function, reduce incidence, and slow progression of AMD, consistently with their abundance in retinal neurons. However, direct assessment of retinal ω‐3 PUFAs content in humans is impossible, making the use of a systemic biomarker a mandatory surrogate for tissue composition. Here, we determine which circulating lipids have the highest predictive performance for retinal content in ω‐3 PUFAs.

As major components of the human retina, ω‐3 PUFAs are essential for retinal physiology. The highest content in ω‐3 PUFAs is found in retinal photoreceptors, where they inhibit several cellular and molecular mechanisms involved in AMD pathogenesis and the related visual loss. While more than 20 epidemiological studies have consistently shown a 40%‐reduction in risk of AMD in subjects with high dietary intake of ω‐3 PUFAs,^2^ and human donor studies have measured lower ω‐3 PUFAs concentration in AMD eyes compared to age‐matched controls,^3^ supplementation trials failed to impact disease progression.^4^, ^5^ This controversy lies partly in the impossibility of directly assess the retinal ω‐3 PUFAs content, making the use of a systemic biomarker a mandatory surrogate for tissue composition.

In the Biomarkers of Lipid Status and Metabolism in Retinal ageing project, we determined which circulating lipids have the highest predictive performance for retinal content in ω‐3 PUFAs. In addition to plasma total lipids, we focused our attention on the longer‐ and medium‐term markers of dietary fat, namely red blood cell phospholipids and plasma phosphatidylcholines and cholesteryl esters. By using human donor samples (Table S1) and advanced lipidomics,^6^ we generated an extremely exhaustive database, corresponding to 304 different lipid species (Tables 1 and S2). Selecting the molecular species relevant for the prediction of retinal ω‐3 PUFAs in this high‐dimensional, low sample size context is not trivial. This is why we tested several methods combining dimension reduction, variable selection, and prior group structure.^7^ The model with the lowest error of prediction was obtained using sparse group partial least squares and was characterized by a cross‐validated r of 0.62 between observed and predicted values of retinal ω‐3 PUFAs content (Figure 1A, see Ajana et al^7^ for details on statistical methods). It was based on an algorithm combining the levels of seven plasma cholesteryl esters.^8^ Three of them were characterized by the presence of PUFAs from ω‐3 family, the four remaining species consisting in a cholesterol molecule esterified to ω‐6 PUFAs (Figure 1B). Interestingly, cholesteryl esters species with ω‐3 PUFAs contributed positively to the estimation of retinal ω‐3 PUFAs content whereas those with ω‐6 PUFAs lowered the prediction index, which is consistent with the well‐established competitive metabolism of ω‐3 and ω‐6 PUFAs. Previous attempts (including ours) on RBC or total plasma lipids have failed in finding strong associations between circulating lipids and absolute levels of retinal ω‐3 PUFAs.^6^, ^9^ Here, we confirm that our predictor is more robust (cross‐validated *r* = 0.62, Figure 1A) than the use of the ω‐3 PUFAs content of RBC (*r* = 0.40) and total plasma (*r* = 0.14).

**TABLE 1:** Fatty acid composition (%) of blood and eye tissues of human donors

	Retina		Red blood cells		Plasma					
					Total		Phosphatidylcholine		Cholesteryl esters	
	Median	*(IQR)*	Median	*(IQR)*	Median	*(IQR)*	Median	*(IQR)*	Median	*(IQR)*
**C14:0**	**0.35**	*(0.13–0.46)*	**0.68**	*(0.32–1.39)*	**0.58**	*(0.43–0.83)*	**0.20**	*(0.11–0.25)*	**0.36**	*(0.16–0.77)*
**C15:0**	**0.10**	*(0.07–0.13)*	**0.23**	*(0.17–0.29)*	**0.22**	*(0.18–0.27)*	**0.23**	*(0.15–0.31)*	**0.20**	*(0015–0.27)*
**C16:0**	**19.65**	*(14.76–21.00)*	**24.34**	*(21.99–26.74)*	**23.42**	*(22.58–25.91)*	**27.15**	*(21.83–32.03)*	**12.67**	*(11.57–14.44)*
**C16:1ω‐9**	**0.75**	*(0.57–0.87)*	**0.30**	*(0.23–0.39)*	**0.30**	*(0.25–0.34)*	**0.16**	*(0.11–0.24)*	**0.33**	*(0.24–0.43)*
**C16:1ω‐7**	**0.55**	*(0.42–0.77)*	**2.30**	*(1.71–2.76)*	**2.72**	*(1.87–3.18)*	**0.48**	*(0.31–0.77)*	**3.27**	*(2.09–4.34)*
**C17:0**	**0.15**	*(0.12–0.18)*	**0.36**	*(0.32–0.44)*	**0.33**	*(0.30–0.36)*	**0.47**	*(0.42–0.57)*	**0.13**	*(0.11–0.17)*
**C18:0**	**20.52**	*(19.40–21.33)*	**9.21**	*(7.55–10.81)*	**6.39**	*(5.73–6.94)*	**13.33**	*(10.92–16.07)*	**0.87**	*(0.73–1.35)*
**C18:1trans**	**0.12**	*(0.08–0.17)*	**0.44**	*(0.35–0.54)*	**0.47**	*(0.31–0.64)*	**0.42**	*(0.27–0.57)*	**0.58**	*(0.20–0.98)*
**C18:1ω‐9**	**15.46**	*(14.12–16.18)*	**29.13**	*(24.93–34.84)*	**29.00**	*(26.52–31.94)*	**15.42**	*(13.07–17.31)*	**25.97**	*(24.06–28.62)*
**C18:1ω‐7**	**3.13**	*(2.88–3.45)*	**2.28**	*(1.96–2.55)*	**2.50**	*(2.22–2.83)*	**2.37**	*(1.99–2.87)*	**2.14**	*(1.83–2.52)*
**C18:2ω‐6**	**1.61**	*(1.35–1.95)*	**12.29**	*(10.30–14.86)*	**19.95**	*(17.40–21.97)*	**16.91**	*(14.68–20.62)*	**41.84**	*(38.03–45.98)*
**C20:0**	**0.53**	*(0.47–0.63)*	**0.17**	*(0.13–0.21)*	**0.14**	*(0.09–0.15)*	**0.17**	*(0.09–0.29)*	**n.d**.	*–*
**C18:3ω‐6**	**0.13**	*(0.11–0.16)*	**n.d**.	*–*	**0.13**	*(0.08–0.22)*	**0.21**	*(0.00–0.26)*	**0.37**	*(0.24–0.61)*
**C20:1ω‐9**	**0.56**	*(0.48–0.68)*	**0.39**	*(0.33–0.49)*	**0.27**	*(0.21–0.32)*	**0.25**	*(0.20–0.45)*	**n.d**.	*–*
**C18:3ω‐3**	**0.15**	*(0.13–0.19)*	**0.30**	*(0.20–0.41)*	**0.37**	*(0.31–0.54)*	**0.18**	*(0.14–0.26)*	**0.45**	*(0.31–0.56)*
**C20:2ω‐6**	**0.16**	*(0.13–0.20)*	**0.22**	*(0.18–0.25)*	**0.19**	*(0.16–0.24)*	**0.38**	*(0.30–0.52)*	**n.d**.	*–*
**C20:3ω‐9**	**0.31**	*(0.21–0.40)*	**n.d**.	*–*	**0.14**	*(0.11–0.19)*	**0.23**	*(0.19–0.33)*	**n.d**.	*–*
**C22:0**	**0.36**	*(0.30–0.49)*	**0.23**	*(0.16–0.32)*	**0.17**	*(0.12–0.21)*	**n.d**.	*–*	**n.d**.	*–*
**C20:3ω‐6**	**1.57**	*(1.29–2.05)*	**0.67**	*(0.42–0.93)*	**0.78**	*(0.59–1.07)*	**2.34**	*(1.78–2.95)*	**0.60**	*(0.45–0.67)*
**C22:1ω‐9**	**0.10**	*(0.07–0.13)*	**n.d**.	*–*	**n.d**.	*–*	**n.d**.	*–*	**n.d**.	*–*
**C20:4ω‐6**	**10.92**	*(10.31–12.15)*	**6.18**	*(3.24–8.12)*	**5.43**	*(4.33–6.79)*	**9.14**	*(6.79–11.44)*	**6.30**	*(4.99–8.34)*
**C24:0**	**0.30**	*(0.25–0.41)*	**0.39**	*(0.27–0.52)*	**0.10**	*(0.00–0.16)*	**n.d**.	*–*	**n.d**.	*–*
**C20:5ω‐3**	**0.18**	*(0.14–0.24)*	**0.28**	*(0.19–0.40)*	**0.43**	*(0.22–0.59)*	**0.63**	*(0.37–0.86)*	**0.62**	*(0.42–0.83)*
**C24:1ω‐9**	**0.11**	*(0.08–0.17)*	**0.64**	*(0.48–0.92)*	**0.42**	*(0.26–0.57)*	**n.d**.	*–*	**n.d**.	*–*
**C22:4ω‐6**	**1.37**	*(1.26–1.62)*	**0.83**	*(0.48–1.12)*	**0.26**	*(0.20–0.30)*	**0.40**	*(0.31–0.45)*	**n.d**.	*–*
**C22:5ω‐6**	**0.53**	*(0.43–0.71)*	**0.17**	*(0.11–0.27)*	**0.15**	*(0.12–0.19)*	**0.27**	*(0.21–0.37)*	**n.d**.	*–*
**C22:5ω‐3**	**1.04**	*(0.86–1.26)*	**0.83**	*(0.40–1.05)*	**0.45**	*(0.38–0.49)*	**1.02**	*(0.70–1.15)*	**n.d**.	*–*
**C22:6ω‐3**	**15.20**	*(13.09–16.87)*	**1.84**	*(0.70–2.31)*	**1.54**	*(1.27–1.89)*	**2.99**	*(2.21–4.20)*	**0.53**	*(0.41–0.67)*
**total SFAs**	**45.34**	*(41.90–46.87)*	**38.41**	*(35.52–40.58)*	**32.40**	*(31.07–34.21)*	**42.63**	*(39.17–45.63)*	**14.80**	*(13.24–17.03)*
**total MUFAs**	**21.49**	*(19.94–22.81)*	**36.48**	*(31.50–29.26)*	**36.42**	*(32.56–39.02)*	**19.48**	*(17.15–21.38)*	**33.14**	*(31.10–35.54)*
**total PUFAs**	**33.41**	*(30.87–36.60)*	**25.25**	*(16.59–29.26)*	**30.70**	*(27.04–34.26)*	**36.86**	*(32.49–40.10)*	**51.7**	*(47.95–54.87)*
**total ω‐6**	**16.42**	*(15.55–19.07)*	**21.32**	*(14.80–24.91)*	**27.48**	*(24.18–30.89)*	**10.75**	*(7.82–12.66)*	**6.90**	*(5.23–8.72)*
**total ω‐3**	**16.79**	*(14.46–18.43)*	**3.41**	*(1.72–4.09)*	**2.89**	*(2.39–3.66)*	**4.95**	*(3.96–6.08)*	**1.63**	*(1.40–2.05)*
**ω‐6 / ω‐3 ratio**	**1.04**	*(0.91–1.20)*	**7.45**	*(5.36–9.28)*	**9.49**	*(7.77–11.33)*	**2.12**	*(1.63–2.76)*	**4.34**	*(3.39–5.51)*

Abbreviations: IQR, interquartile range; MUFAs, monounsaturated fatty acids; n.d., not detected; PUFAs, polyunsaturated fatty acids; SFAs, saturated fatty acids.

**FIGURE 1:**
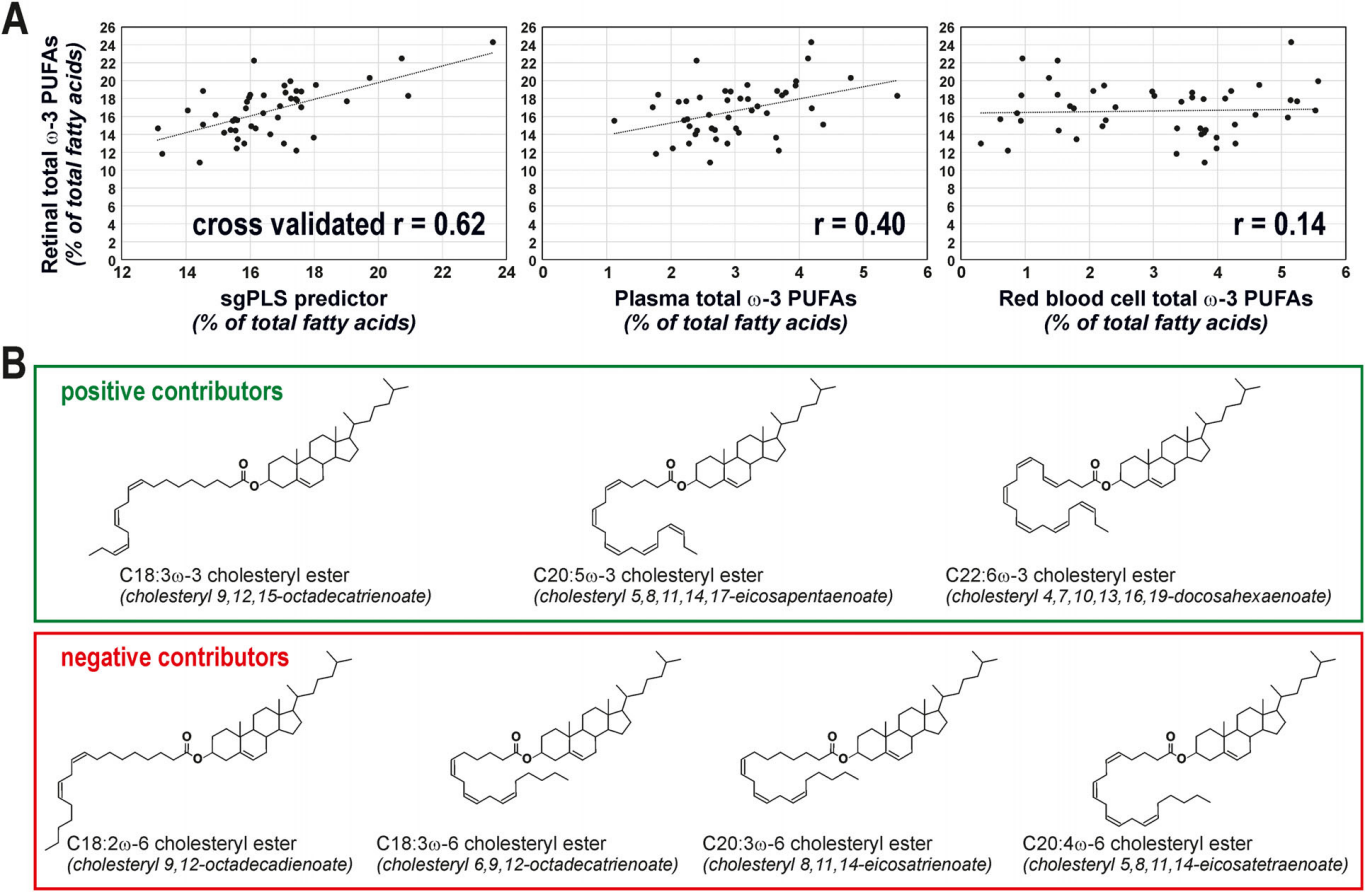
Plasma cholesteryl esters as markers of retinal content in ω‐3 PUFAs. (A) Output from the prediction model resulting from sgPLS analysis showing a higher robustness of predictor when compared to total plasma and RBC ω‐3 PUFAs in estimating retinal ω‐3 PUFA content. (B) Molecular species of cholesteryl esters whose plasma concentration values are used to predict retinal ω‐3 PUFA content. The plasma concentration values of cholesteryl esters with ω‐3 PUFAs contributed positively to the calculated index whereas those of ω‐6 PUFA cholesteryl esters lowered it

In the second phase, we further explored the associations of AMD with ω‐3 PUFAs status. First, in a postmortem case‐control study, we confirmed previous data showing a lower ω‐3 PUFAs content in retinas affected by AMD when compared to those from healthy donors (Figure 2).^3^, ^9^ After statistical adjustments (Table S3), the difference between AMD and control retinas was of ‐2.41% of total fatty acids. Then, using the algorithm based on seven plasma cholesteryl esters developed at phase 1, we compared the predicted retinal ω‐3 PUFAs content between 31 subjects affected by advanced AMD and 31 controls (Table 2) and found that it was lower in cases compared to controls. The difference in predicted retinal ω‐3 PUFAs content between AMD cases and controls was of −1.39%. These observations are consistent with the pathophysiology of AMD that is characterized by the loss of the ω‐3 PUFA‐rich photoreceptor cells.

**FIGURE 2:**
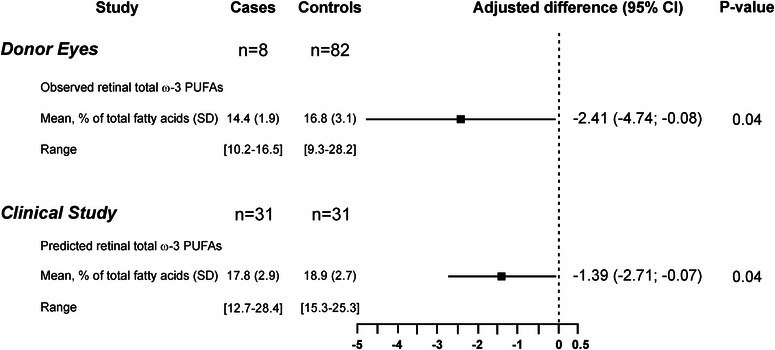
Predicted levels of retinal ω‐3 PUFAs are discriminant for AMD and equivalent to a direct assessment: case‐control studies. Adjusted differences black squares and 95% confidence intervals (CIs) (horizontal lines) of observed and predicted retinal ω‐3 PUFAs contents for age‐related macular degeneration in donor eyes and humans patients. For postmortem study, adjusted difference was assessed by linear regression adjusted for age, gender, and time after death. For clinical study, adjusted difference was assessed by mixed linear regression adjusted for age, body mass index, smoking, ω‐3 supplement use, HDL‐cholesterol, and LDL‐cholesterol

**TABLE 2:** Characteristics of the participants to the case‐control and LIMPIA studies

Case‐control study	cases (*n* = 31)	controls (*n* = 31)	*p* value
Age, mean (SD) (range), y	84.5 (4.2) (76.7–92.5)	84.2 (4.3) (76.8–90.7)	0.4
Female, no (%)	22 (71.0)	22 (71.0)	–
BMI, mean (SD), kg/m²	24.8 (3.0) (18.9–31.6)	24.8 (3.6) (18.1–34.7)	0.98
Plasma total cholesterol, mean (SD) (range), mmol/L	5.89 (0.98) (3.32–8.62)	5.59 (1.00) (3.00–7.56)	0.24
Plasma HDL cholesterol, mean (SD) (range), mmol/L	1.62 (0.34) (1.01–2.18)	1.45 (0.38) (0.82–2.22)	0.05
Plasma LDL cholesterol, mean (SD) (range), mmol/L	3.73 (0.84) (1.67–6.01)	3.57 (0.94) (1.14–5.55)	
Plasma triglycerides, mean (SD) (range), mmol/L	1.19 (0.53) (0.69–3.22)	1.26 (0.56) (0.40–2.37)	0.49
ω‐3 Supplement use, yes (%)	6 (19.4)	1 (3.2)	0.10
Smoking, no (%)			0.55
Never smoker	19 (61.3)	21 (67.7)	
<20 pack‐year	5 (16.1)	6 (19.3)	
≥20pack‐year	7 (22.6)	4 (13.0)	

LIMPIA study	Supplemented (*n* = 29)	Placebo (*n* = 26)	*p* value
Age, mean (SD) (range), y	57.7 (6.3) (44.6–70.2)	55.6 (6.9) (44.3–66.1)	0.22
Female, no (%)	22 (75.9)	20 (76.9)	0.93
BMI, mean (SD), kg/m²	24.2 (3.5) (19.2–31.3)	23.8 (3.8) (18.1–32.8)	0.74
Plasma total cholesterol, mean (SD) (range), mmol/L	5.85 (0.81) (4.32–7.62)	5.84 (0.72) (4.28–7.53)	0.95
Plasma HDL cholesterol, mean (SD) (range), mmol/L	1.73 (0.37) (1.14–2.54)	1.74 (0.56) (0.98–3.44)	0.94
Plasma LDL cholesterol, mean (SD) (range), mmol/L	3.58 (0.77) (1.74–4.98)	3.58 (0.73) (1.37–4.82)	0.99
Plasma triglycerides, mean (SD) (range), mmol/L	1.19 (0.44) (0.61–2.16)	1.14 (0.42) (0.50–1.98)	0.68
Smoking, no (%)			0.85
Never smoker	16 (61.5)	16 (55.2)	
<20 pack‐year	6 (23.1)	7 (24.1)	
≥20pack‐year	4 (15.4)	6 (20.7)	

Abbreviations: BMI, body mass index; HDL, high density lipoprotein; LDL, low density lipoprotein; SD, standard deviation.

Considering the crucial functions played by ω‐3 PUFAs in retinal structure and function and its depletion in AMD eyes, the idea that maintaining high retinal levels of ω‐3 PUFAs would protect against the development and/or the progression of the disease makes sense. This hypothesis is consolidated by a large number of studies showing a significantly reduced risk for developing AMD in subjects with high dietary consumption of ω‐3 PUFAs.^2^ However, in the two randomized trials AREDS2 and NAT2,^4^, ^5^ ω‐3 PUFAs supplementation did not modify disease progression. One possible explanation to these contradictory results lies on subjects’ sensitivity to nutritional supplementation. Indeed, whereas ω‐3 PUFAs supplementation had no effect on AMD progression when considering total population, we have shown in NAT2 study that subjects who maintained steadily high blood levels of ω‐3 PUFAs had a significantly lower risk for developing advanced AMD.^10^ This finding confirms the need of monitoring the metabolic status of subjects participating in nutritional interventions. This is why we evaluated the sensitivity of the predicted retinal ω‐3 PUFAs content to a dietary supplementation with ω‐3 PUFAs. We analyzed blood samples collected within the LIMPIA randomized clinical trial in which participants received ω‐3 PUFAs for 6 months (Table 2). Whereas the median values of predicted retinal ω‐3 PUFAs content at baseline were similar in both groups, they were significantly increased in supplemented subjects at 3 months, this difference being maintained after 6 months of supplementation (Figure 3).

**FIGURE 3:**
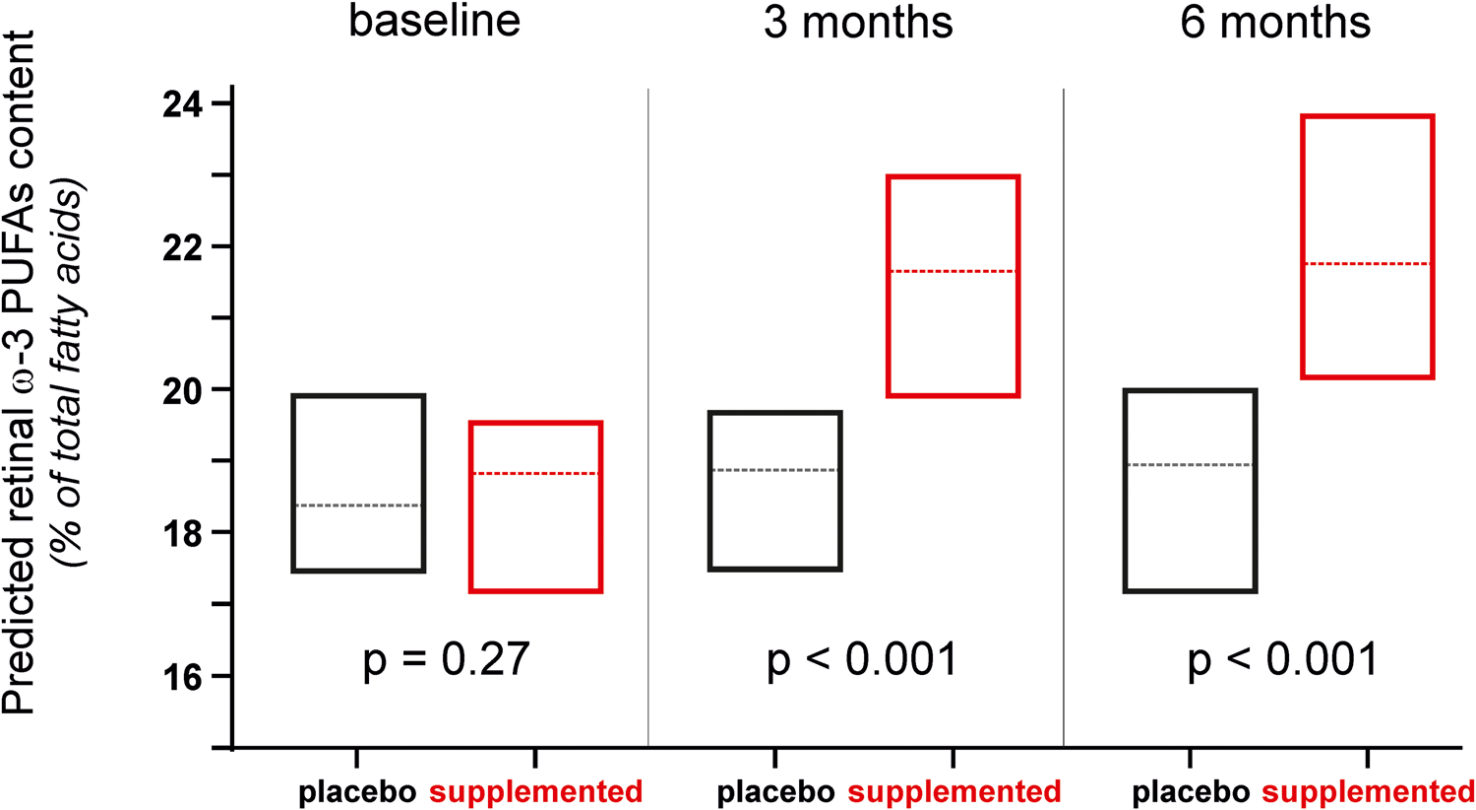
Predicted levels of retinal ω‐3 PUFAs are sensitive to a dietary supplementation with ω‐3 PUFAs. Medians (horizontal lines) and interquartile ranges (boxes) of predicted retinal ω‐3 PUFAs content among participants to the LIMPIA study. Linear regression model adjusted for age, gender, body mass index, smoking, plasma LDL‐cholesterol, and plasma HDL‐cholesterol

In summary, we identified a blood biomarker of retinal ω‐3 PUFAs status, which is inversely related to the risk for advanced AMD and increased with supplementation in ω‐3 PUFAs. If confirmed in larger studies, it may serve as a practical tool for scientists to set‐up and conduct clinical trials, but also to help doctors to diagnose the condition earlier and single out individuals who are more likely to benefit from nutritional treatment. Finally, an independent model validation dataset and samples from intermediate‐AMD subjects would also strengthen our findings.

## CONFLICT OF INTEREST

The authors declare no conflict of interest related to this study.

## AUTHOR CONTRIBUTIONS

Designed the studies: Niyazi Acar, Benjamin Buaud, Lionel Bretillon, and Cécile Delcourt. Conducted experiments: Zhiguo He, Stéphane Grégoire, Lucy Martine, and Benjamin Buaud. Acquired data: Stéphane Grégoire, Lucy Martine, Benjamin Buaud, Olivier Berdeaux, Jean‐François Korobelnik, Catherine P. Creuzot‐Garcher, and Alain M. Bron. Analyzed data: Niyazi Acar, Bénédicte M. J. Merle, Soufiane Ajana, Boris P. Hejblum, Benjamin Buaud, Olivier Berdeaux, Hélène Jacqmin‐Gadda, Lionel Bretillon, and Cécile Delcourt. Wrote manuscript: Niyazi Acar, Bénédicte M. J. Merle, Soufiane Ajana, Zhiguo He, Stéphane Grégoire, Boris P. Hejblum, Lucy Martine, Benjamin Buaud, Alain M. Bron, Catherine P. Creuzot‐Garcher, Jean‐François Korobelnik, Olivier Berdeaux, Hélène Jacqmin‐Gadda, Lionel Bretillon, and Cécile Delcourt.

## AVAILABILITY OF DATA AND MATERIALS

The datasets generated and analyzed during the current study are neither publicly available due to personal information involved nor from the corresponding author due to a pending patent.

## Supporting information

SUPPORTING INFORMATION

SUPPORTING INFORMATION

SUPPORTING INFORMATION
