# Supplementary material for: Predicting the retinal content in omega‐3 fatty acids for age‐related macular‐degeneration
Source: Clin Transl Med. 2021 Jun 30;11(7):e404. doi: 10.1002/ctm2.404 (PMC8243522; doi:10.1002/ctm2.404)
Supplement: Supplementary file 1 — SUPPORTING INFORMATION [file CTM2-11-e404-s003.docx]

**Table S1.** Characteristics of the human donors

| **subject** | **gender** | **age (yr)** | **delay of collection after death (h)** |
| --- | --- | --- | --- |
| #1 | male | 84 | 34.0 |
| #2 | male | 69 | 11.0 |
| #3 | female | 93 | 13.0 |
| #4 | female | 73 | 5.0 |
| #5 | female | 83 | 18.0 |
| #6 | male | 98 | 18.0 |
| #7 | male | 67 | 11.0 |
| #8 | male | 93 | 12.0 |
| #9 | female | 83 | 16.0 |
| #10 | female | 81 | 10.0 |
| #11 | female | 89 | 10.0 |
| #12 | female | 73 | 8.0 |
| #13 | male | 81 | 42.0 |
| #14 | female | 97 | 35.0 |
| #15 | female | 92 | 5.0 |
| #16 | female | 85 | 20.0 |
| #17 | female | 92 | 26.0 |
| #18 | female | 69 | 16.0 |
| #19 | female | 94 | 4.0 |
| #20 | female | 81 | 12.0 |
| #21 | female | 89 | 28.0 |
| #22 | female | 94 | 21.0 |
| #23 | male | 75 | 30.0 |
| #24 | female | 86 | 30.0 |
| #25 | male | 70 | 24.0 |
| #26 | female | 63 | 21.0 |
| #27 | male | 95 | 11.0 |
| #28 | male | 79 | 23.0 |
| #29 | male | 79 | 22.0 |
| #30 | female | 87 | 10.0 |
| #31 | female | 89 | 29.0 |
| #32 | female | 95 | 4.0 |
| #33 | female | 92 | 21.0 |
| #34 | female | 87 | 2.5 |
| #35 | male | 92 | 14.0 |
| #36 | male | 75 | 10.0 |
| #37 | female | 84 | 6.0 |
| #38 | female | 101 | 33.0 |
| #39 | female | 91 | 9.0 |
| #40 | female | 91 | 14.0 |
| #41 | male | 92 | 7.0 |
| #42 | male | 71 | 17.0 |
| #43 | female | 89 | 8.0 |
| #44 | female | 55 | 21.0 |
| #45 | female | 88 | 4.0 |
| #46 | male | 63 | 10.0 |
| **mean ± SD** | - | 83.7 ± *10.7* | 16.4 ± *9.7* |
| **median** | - | 86.5 | 10.0 |
| ***[IQR]*** |  | *[76-92]* | *[10.0-21.8]* |
