# Supplementary material for: Predicting the retinal content in omega‐3 fatty acids for age‐related macular‐degeneration
Source: Clin Transl Med. 2021 Jun 30;11(7):e404. doi: 10.1002/ctm2.404 (PMC8243522; doi:10.1002/ctm2.404)
Supplement: Supplementary file 2 — SUPPORTING INFORMATION [file CTM2-11-e404-s002.docx]

**Table S2.** Concentrations of individual phospholipid species of red blood cells of human donors (µg/mg of total phospholipids)

|  |  | **Red Blood cells** | |
| --- | --- | --- | --- |
|  | **[M+H]+ or MS/MS transition*^a^*** | **median** | *[IQR]* |
| PC 30:0 | 706.40 | **1.38** | *[1.24–1.83]* |
| PlsC 32:0 | 718.60 | **0.53** | *[0.36-0.97]* |
| PC 32:1 | 720.60 | **2.24** | *[1.89-2.95]* |
| PC 32:2 | 730.53 | **0.42** | *[0.35-0.56]* |
| PC 32:1 | 732.50 | **6.00** | *[4.67-8.15]* |
| PC 32:0 | 734.56 | **14.69** | *[13.23-16.27]* |
| PlsC 34:2 | 742.47 | **0.25** | *[0.16-0.82]* |
| PlsC 34:1 | 744.58 | **1.92** | *[1.11-2.49]* |
| PlsC 34:0 | 746.50 | **4.93** | *[4.12-6.07]* |
| PC 34:4 | 754.60 | **0.18** | *[0.14-0.21]* |
| PC 34:3 | 756.60 | **1.79** | *[1.54-2.45]* |
| PC 34:2 | 758.60 | **78.94** | *[57.91-104.09]* |
| PC 34:1 | 760.60 | **94.85** | *[85.18-114.54]* |
| PC 34:0 | 762.60 | **2.92** | *[2.32-3.69]* |
| PlsC 36:5 | 764.60 | **0.36** | *[0.24-0.58]* |
| Pls C 36:4 | 766.60 | **1.38** | *[0.00-2.63]* |
| PlsC 36:3 | 768.58 | **2.86** | *[0.94-3.88]* |
| PlsC 36:2 | 770.60 | **1.41** | *[1.20-1.90]* |
| PlsC 36:1 | 772.61 | **4.53** | *[4.15-5.19]* |
| PlsC 36:0 | 774.60 | **4.88** | *[3.71-5.54]* |
| PC 36:5 | 780.60 | **1.96** | *[1.49-3.21]* |
| PC 36:4 | 782.63 | **24.59** | *[18.16-35.61]* |
| PC 36:3 | 784.63 | **16.76** | *[13.18-19.79]* |
| PC 36:2 | 786.63 | **33.45** | *[28.88-41.10]* |
| PC 36:1 | 788.34 | **16.67** | *[14.81-19.10]* |
| PC 36:0 and/or PlsC 38:6 | 790.70 | **0.97** | *[0.70-1.21]* |
| PlsC 38:5 | 792.70 | **1.21** | *[0.78-1.44]* |
| PlsC 38:4 | 794.70 | **3.04** | *[2.00-3.58]* |
| PlsC 38:3 | 796.70 | **2.08** | *[0.00-3.19]* |
| PlsC 38:2 | 798.63 | **1.20** | *[1.02-1.59]* |
| PlsC 38:0 | 802.66 | **1.10** | *[0.86-1.40]* |
| PC 38:7 | 804.60 | **0.54** | *[0.42-0.85]* |
| PC 38:6 | 806.58 | **9.83** | *[5.75-13.85]* |
| PC 38:5 | 808.00 | **6.98** | *[5.02-8.42]* |
| PC 38:4 | 810.60 | **13.10** | *[9.41-16.32]* |
| PC 38:3 | 812.60 | **3.87** | *[2.90-5.03]* |
| PC 38:2 | 814.60 | **1.45** | *[1.27-1.72]* |
| PlsC 40:7 | 816.35 | **1.20** | *[0.88-1.59]* |
| PlsC 40:6 | 818.63 | **0.86** | *[0.68-1.03]* |
| PlsC 40:5 | 820.61 | **1.05** | *[0.73-1.29]* |
| PlsC 40:4 | 822.68 | **0.95** | *[0.83-1.12]* |
| PlsC 40:3 | 824.68 | **0.63** | *[0.50-0.79]* |
| PlsC 40:2 | 826.58 | **0.50** | *[0.36-0.69]* |
| PlsC 40:1 | 828.00 | **0.52** | *[0.30-0.64]* |
| PC 40:2 | 830.60 | **0.57** | *[0.43-0.72]* |
| PC 40:7 | 832.60 | **0.94** | *[0.74-1.10]* |
| PC 40:6 | 834.68 | **3.07** | *[2.31-4.15]* |
| PC 40:5 | 836.60 | **1.53** | *[1.26-1.98]* |
| PC 40:4 | 838.63 | **0.70** | *[0.56-0.83]* |
| PC 40:3 | 840.64 | **0.08** | *[0.00-0.18]* |
| PC 40:2 | 842.60 | **0.19** | *[0.12-0.31]* |
| PC40:1 | 844.70 | **0.23** | *[0.16-0.37]* |
| PC 40:0 | 846.00 | **0.23** | *[0.17-0.46]* |
| PC 42:12 | 850.50 | **0.30** | *[0.00-0.55]* |
| PC 42:9 | 856.60 | **0.47** | *[0.31-0.75]* |
| PC 42:6 | 862.62 | **0.12** | *[0.09-0.22]* |
| PC 42:5 | 864.00 | **0.17** | *[0.09-0.29]* |
| PC 42:2 | 870.00 | **0.03** | *[0.00-0.10]* |
| PC 42:1 | 872.70 | **0.17** | *[0.15-0.22]* |
| PC 44:12 | 878.60 | **0.43** | *[0.27-0.71]* |
| PC 44:11 | 880.60 | **0.13** | *[0.09-0.17]* |
| PC 44:10 | 882.60 | **0.07** | *[0.03-0.09]* |
| PC 44:9 | 884.60 | **0.07** | *[0.01-0.11]* |
| PC 44:8 | 886.60 | **0.02** | *[0.00-0.04]* |
| PC 45:10 | 896.60 | **0.00** | *[0.00-0.02]* |
| PC 45:9 | 898.00 | **0.00** | *[0.00-0.04]* |
| PC 45:8 | 900.64 | **0.03** | *[0.00-0.09]* |
| PC 46:12 | 906.60 | **0.07** | *[0.05-0.12]* |
| PC 46:11 | 908.70 | **0.01** | *[0.00-0.02]* |
| PC 46:10 | 910.70 | **0.00** | *[0.00-0.00]* |
| PC 46:9 | 912.60 | **0.00** | *[0.00-0.00]* |
| PC 47:12 | 920.60 | **0.00** | *[0.00-0.00]* |
| PC 47:11 | 922.60 | **0.00** | *[0.00-0.00]* |
| PC 47:9 | 926.70 | **0.00** | *[0.00-0.00]* |
| PC 47:8 | 928.70 | **0.00** | *[0.00-0.01]* |
| PC 48:7 | 944.70 | **0.04** | *[0.00-0.06]* |
| PC 52:11 | 992.80 | **0.00** | *[0.00-0.01]* |
| PC 52:10 | 994.80 | **n.d.** | *-* |
| PC 52:7 | 1000.70 | **n.d.** | *-* |
| PC 52:6 | 1002.70 | **n.d.** | *-* |
| PC 54:12 | 1018.77 | **n.d.** | *-* |
| PC 54:11 | 1020.77 | **n.d.** | *-* |
| PC 54:10 | 1022.82 | **n.d.** | *-* |
| PC 54:9 | 1024.80 | **n.d.** | *-* |
| PC 56:12 | 1046.82 | **n.d.** | *-* |
| PC 56:11 | 1048.82 | **n.d.** | *-* |
| PC 56:10 | 1050.82 | **n.d.** | *-* |
| PC 56:9 | 1052.80 | **n.d.** | *-* |
| PC 58:12 | 1074.82 | **n.d.** | *-* |
| PC 58:11 | 1076.87 | **n.d.** | *-* |
| Total PC + PlsC | - | **401.75** | *[366.15-445.45]* |
| PE 30:3 | 658.40 | **0.05** | *[0.30-0.10]* |
| PE 32:1 | 690.53 | **0.50** | *[0.39-0.73]* |
| PE 32:0 | 692.54 | **0.66** | *[0.53-0.85]* |
| PE 33:0 | 706.53 | **0.11** | *[0.06-0.16]* |
| PE 34:3 | 714.50 | **0.42** | *[0.27-0.68]* |
| PE 34:2 | 716.54 | **7.24** | *[5.37-10.02]* |
| PE 34:1 | 718.56 | **25.41** | *[19.60-29.54]* |
| PE 34:0 | 720.57 | **0.00** | *[0.00-0.00]* |
| PE 36:6 | 736.48 | **0.08** | *[0.04-0.13]* |
| PE 36:5 | 738.50 | **1.03** | *[0.49-1.30]* |
| PE 36:4 | 740.54 | **10.35** | *[8.05-16.21]* |
| PE 36:3 | 742.56 | **5.87** | *[3.27-7.80]* |
| PE 36:2 | 744.57 | **12.14** | *[8.61-14.07]* |
| PE 36:1 | 746.59 | **8.88** | *[6.58-13.01]* |
| PE 36:0 | 748.55 | **0.00** | *[0.00-0.00]* |
| PE 38:7 | 762.50 | **0.46** | *[0.25-0.91]* |
| PE 38:6 | 764.54 | **8.27** | *[5.30-10.96]* |
| PE 38:5 | 766.56 | **10.05** | *[6.56-16.17]* |
| PE 38:4 | 768.57 | **15.29** | *[11.31-22.96]* |
| PE 38:3 | 770.59 | **0.95** | *[0.62-1.41]* |
| PE 38:2 | 772.60 | **0.49** | *[0.31-0.85]* |
| PE 38:1 | 774.56 | **0.25** | *[0.09-0.46]* |
| PE 38:0 | 776.50 | **0.26** | *[0.13-0.40]* |
| PE 40:9 | 786.50 | **0.28** | *[0.19-0.47]* |
| PE 40:8 | 788.54 | **0.70** | *[0.28-0.96]* |
| PE 40:7 | 790.56 | **3.65** | *[2.26-4.33]* |
| PE 40:6 | 792.57 | **4.44** | *[2.83-5.76]* |
| PE 40:5 | 794.59 | **3.47** | *[2.44-4.60]* |
| PE 40:4 | 796.60 | **1.73** | *[1.17-2.48]* |
| PE 40:3 | 798.62 | **0.01** | *[0.00-0.09]* |
| PE 40:2 | 800.65 | **0.14** | *[0.10-0.27]* |
| PE 40:1 | 802.62 | **0.08** | *[0.01-0.14]* |
| PE 40:0 | 804.64 | **0.09** | *[0.00-0.29]* |
| PE 42:13 | 806.60 | **0.10** | *[0.03-0.15]* |
| PE 42:12 | 808.00 | **0.11** | *[0.08-0.17]* |
| PE 42:11 | 810.00 | **0.07** | *[0.00-0.16]* |
| PE 42:10 | 812.52 | **0.18** | *[0.00-0.36]* |
| PE 42:9 | 814.56 | **0.23** | *[0.13-0.33]* |
| PE 42:8 | 816.50 | **0.21** | *[0.09-0.32]* |
| PE 42:7 | 818.56 | **0.22** | *[0.09-0.30]* |
| PE 42:6 | 820.55 | **0.14** | *[0.08-0.18]* |
| PE 42:5 | 822.62 | **0.06** | *[0.00-0.16]* |
| PE 42:4 | 824.63 | **0.06** | *[0.00-0.08]* |
| PE 42:3 | 826.00 | **0.04** | *[0.00-0.07]* |
| PE 42:2 | 828.64 | **0.05** | *[0.00-0.07]* |
| PE 42:0 | 832.67 | **0.02** | *[0.00-0.05]* |
| inconnu | 834.00 | **0.02** | *[0.01-0.04]* |
| PE 44:12 | 836.56 | **0.03** | *[0.00-0.06]* |
| PE 44:11 | 838.60 | **0.02** | *[0.00-0.04]* |
| PE 44:10 | 840.50 | **0.02** | *[0.00-0.05]* |
| PE 44:9 | 842.60 | **0.02** | *[0.00-0.05]* |
| PE 44:8 | 844.60 | **0.00** | *[0.00-0.02]* |
| unknown | 846.00 | **0.00** | *[0.00-0.00]* |
| unknown | 850.00 | **0.00** | *[0.00-0.00]* |
| unknown | 852.00 | **0.00** | *[0.00-0.00]* |
| unknown | 854.00 | **0.00** | *[0.00-0.00]* |
| unknown | 856.00 | **0.00** | *[0.00-0.00]* |
| unknown | 862.00 | **0.00** | *[0.00-0.00]* |
| PlsE 16:0/20:4 | 722->303 | **0.13** | *[0.00-2.92]* |
| PlsE 16:0/20:3 | 724->305 | **0.01** | *[0.00-0.29]* |
| PlsE 18:0/18:1 | 728->281 | **0.32** | *[0.15-2.71]* |
| PlsE 16:0/22:6 | 746->327 | **0.01** | *[0.00-0.27]* |
| PlsE 18:1/20:4 | 748->303 | **0.18** | *[0.00-2.26]* |
| PlsE 16:0/22:5 | 748->329 | **0.05** | *[0.00-1.55]* |
| PlsE 18:0/20:4 | 750->303 | **0.68** | *[0.24-7.84]* |
| PlsE 16:0/22:4 | 750->331 | **0.14** | *[0.00-2.41]* |
| PlsE 18:1/20:2 | 752->307 | **0.00** | *[0.00-0.02]* |
| PlsE 18:1/20:1 | 754->309 | **0.01** | *[0.00-0.08]* |
| PlsE 18:1/22:6 | 772->327 | **0.01** | *[0.00-0.24]* |
| PlsE 18:0/22:6 | 774->327 | **0.06** | *[0.01-0.87]* |
| PlsE 18:1/22:4 | 776->331 | **0.08** | *[0.00-0.76]* |
| PlsE 18:0/22:5 | 776->329 | **0.19** | *[0.06-2.03]* |
| PlsE 18:1/22:5 | 778->329 | **0.35** | *[0.20-0.43]* |
| Total PE+PlsE | - | **150.54** | *[102.27-185.59]* |

*^a^*: [M+H]+ for PC, PlsC, and PE species and MS/MS transition for PlsE species

IQR: interquartile range; n.d.: not detected; SFAs: saturated fatty acids; MUFAs: monounsaturated fatty acids; PUFAs: polyunsaturated fatty acids.

PC: phosphatidylcholine; PlsC: plasmenylcholine; PE: phosphatidylethanolamine: PlsE: plasmenylethanolamine

n.d.: not detected

Abbreviations of individual PC, PlsC, PE, and PlsE species are as follows: position on the glycerol backbone as shown as sn-1/sn-2 of the fatty acid and fatty alcohol radicals (abbreviated as number of carbons: number of double bonds).
