# Supplementary material for: Predicting the retinal content in omega‐3 fatty acids for age‐related macular‐degeneration
Source: Clin Transl Med. 2021 Jun 30;11(7):e404. doi: 10.1002/ctm2.404 (PMC8243522; doi:10.1002/ctm2.404)
Supplement: Supplementary file 3 — SUPPORTING INFORMATION [file CTM2-11-e404-s001.docx]

**Table S3.** Univariate (unadjusted) and multivariate (adjusted) statistical models of case-control studies

|  | **non-adjusted difference** | | | **adjusted difference** | | |
| --- | --- | --- | --- | --- | --- | --- |
| **Donor eyes*** | **difference** | **95%CI** | **P-value** | **difference** | **95%CI** | **P-value** |
| Observed retinal  ω-3 PUFAs | -2.33 | (-4.58; 0.07) | 0.04 | -2.41 | (-4.74; -0.08) | 0.04 |
|  |  |  |  |  |  |  |
| Age |  |  |  | -0.09 | (-0.16; -0.02) | 0.01 |
|  |  |  |  |  |  |  |
| Gender |  |  |  | -0.03 | (-1.40; 1.34) | 0.96 |
|  |  |  |  |  |  |  |
| Delay of collection after death |  |  |  | -0.04 | (-0.11; 0.04) | 0.34 |
|  |  |  |  |  |  |  |
| **Clinical study**** | **difference** | **95%CI** | **P-value** | **difference** | **95%CI** | **P-value** |
| Predicted retinal  ω-3 PUFAS | -1.07 | (-2.40; 0.26) | 0.11 | -1.39 | (-2.71; -0.07) | 0.04 |
|  |  |  |  |  |  |  |
| Age |  |  |  | -0.09 | (-0.28; 0.09) | 0.30 |
|  |  |  |  |  |  |  |
| BMI |  |  |  | -0.10 | (-0.33; 0.13) | 0.37 |
|  |  |  |  |  |  |  |
| Smoking: |  |  |  |  |  | 0.91 |
| never smoker |  |  |  | ref |  |  |
| <20 pack-year |  |  |  | 0.32 | (-1.57; 2.20) |  |
| ≥20 pack-year |  |  |  | 0.31 | (-1.63; 2.25) |  |
|  |  |  |  |  |  |  |
| ω-3 supplement user |  |  |  | 1.96 | (-0.45; 4.38) | 0.11 |
|  |  |  |  |  |  |  |
| Plasma LDL cholesterol, |  |  |  | 0.40 | (-1.83; 2.64) | 0.71 |
|  |  |  |  |  |  |  |
| Plasma HDL cholesterol |  |  |  | -0.38 | (-1.17; 0.42) | 0.33 |

BMI: Body Mass Index; HDL: High Density Lipoprotein; LDL: Low Density Lipoprotein

*linear regression

** mixed linear regression with a random factor for the case-control pairs.
